# Development and internal validation of a risk model for hyperuricemia in diabetic kidney disease patients

**DOI:** 10.3389/fpubh.2022.863064

**Published:** 2022-10-19

**Authors:** Guoqing Huang, Mingcai Li, Yushan Mao, Yan Li

**Affiliations:** ^1^Department of Endocrinology, The Affiliated Hospital of Medical School, Ningbo University, Ningbo, China; ^2^School of Medicine, Ningbo University, Ningbo, China

**Keywords:** diabetic kidney disease, hyperuricemia, independent risk factors, prediction model, nomogram

## Abstract

**Purpose:**

This research aimed to identify independent risk factors for hyperuricemia (HUA) in diabetic kidney disease (DKD) patients and develop an HUA risk model based on a retrospective study in Ningbo, China.

**Patients and methods:**

Six hundred and ten DKD patients attending the two hospitals between January 2019 and December 2020 were enrolled in this research and randomized to the training and validation cohorts based on the corresponding ratio (7:3). Independent risk factors associated with HUA were identified by multivariable logistic regression analysis. The characteristic variables of the HUA risk prediction model were screened out by the least absolute shrinkage and selection operator (LASSO) combined with 10-fold cross-validation, and the model was presented by nomogram. The C-index and receiver operating characteristic (ROC) curve, calibration curve and Hosmer–Lemeshow test, and decision curve analysis (DCA) were performed to evaluate the discriminatory power, degree of fitting, and clinical applicability of the risk model.

**Results:**

Body mass index (BMI), HbA1c, estimated glomerular filtration rate (eGFR), and hyperlipidemia were identified as independent risk factors for HUA in the DKD population. The characteristic variables (gender, family history of T2DM, drinking history, BMI, and hyperlipidemia) were screened out by LASSO combined with 10-fold cross-validation and included as predictors in the HUA risk prediction model. In the training cohort, the HUA risk model showed good discriminatory power with a C-index of 0.761 (95% CI: 0.712–0.810) and excellent degree of fit (Hosmer–Lemeshow test, *P* > 0.05), and the results of the DCA showed that the prediction model could be beneficial for patients when the threshold probability was 9–79%. Meanwhile, the risk model was also well validated in the validation cohort, where the C-index was 0.843 (95% CI: 0.780–0.906), the degree of fit was good, and the DCA risk threshold probability was 7–100%.

**Conclusion:**

The development of risk models contributes to the early identification and prevention of HUA in the DKD population, which is vital for preventing and reducing adverse prognostic events in DKD.

## Introduction

Diabetes mellitus (DM) is a metabolic disease caused by a combination of genetic, environmental factors and dietary habits and is characterized by chronic elevation of blood glucose and inadequate insulin secretion. With the prolonged course of DM and poor long-term glycemic control, the accumulation of abnormal substances in the metabolic process (such as advanced glycosylation end products, free fatty acids, and inflammation-related mediators) can cause functional damage to multiple organs of the body, including the kidneys, retina, and heart and brain vessels. Among them, diabetic kidney disease (DKD) and cardiovascular disease are the leading causes of death and disability in diabetic patients, posing a significant threat to human physical and mental health.

DKD is one of the most important microvascular complications of DM, with an increased urinary albumin excretion rate and reduced glomerular filtration rate as the main clinical manifestations (1). The main pathological changes of DKD are proliferation of thylakoid cells, extracellular matrix accumulation, basement membrane thickening, diffuse glomerulosclerosis, and interstitial fibrosis (2, 3). In recent years, as the prevalence of DM has increased globally, the prevalence of DKD has also increased, with approximately 40% of DM patients suffering from DKD, which is a significant cause of chronic kidney disease (CKD) and end-stage renal disease (ESRD) (4). Uric acid (UA) is the end product of the metabolism of purine compounds with a dynamic balance of production and clearance under normal conditions. However, the disruption of the balance inevitably causes a continuous increase in UA levels, which in turn results in the development of hyperuricemia (HUA). The kidney plays an important role in the excretion of uric acid, of which approximately 90% of HUA is the result of abnormal glomerular and/or tubular function (5).

The public has increasingly recognized HUA as a risk factor for DKD (6–8), and UA may become a new therapeutic target for DKD. However, other studies have shown no causal relationship between elevated UA levels and kidney disease only as a downstream marker of kidney damage (9, 10). Few studies on risk factors for HUA in the DKD population have been reported. There have been many studies on HUA risk prediction models, but most were developed based on healthy populations. Cao et al. (11) developed a simple HUA Cox proportional hazard model based on an urban Chinese population that showed good clinical discrimination between men and women [C-index: 0.783 (95% CI: 0.779–0.786) vs. 0.784 (95% CI: 0.778–0.789)]. Gao et al. (12) constructed a random forest prediction model for health checkups. In addition, risk prediction models based on machine learning, such as artificial neural networks, are also used for HUA prediction (13, 14). The predictive model is established to serve the clinic better, so the characteristics of solid predictive ability, visualization, and easy operation are necessary. The least absolute shrinkage and selection operator (LASSO) combined with 10-fold cross-validation was used to screen for characteristic variables, while the nomogram provides a tool for the visual representation of predictive models. The establishment of HUA risk prediction would contribute to the early intervention of DKD, the delay of the disease course, and the reduction of adverse prognostic events. The purpose of this study was, on the one hand, to identify independent risk factors for HUA in the DKD population and, on the other hand, to develop a risk model for HUA with the help of the nomogram.

## Materials and methods

### Patients

From January 2019 to December 2020, questionnaires were administered to T2DM patients who were outpatients and inpatients in two hospitals in Ningbo, including the Affiliated Hospital of Medical School, Ningbo University, Yinzhou No. 3 Hospital. Relevant clinical data were obtained and recorded through questionnaires, physical examinations, and laboratory tests. To ensure the accuracy of the study, the completeness of each individual data was checked, those with more missing values (exceeding 20% of the total) were removed, and those with fewer missing values (< 20% of the total) were filled with multiple imputation (15). After data processing, complete information was obtained for 1,682 T2DM patients. Finally, 610 patients with clearly diagnosed DKD were included in the study by reviewing past medical history and inquiry. The diagnosis of DKD meets one of the following criteria (16): (1) random urine albumin creatinine ratio (ACR) ≥30 mg/g or urinary albumin excretion rate ≥30 mg/24 h, and the critical value is reached or exceeded in two out of three tests within 3 to 6 months; (2) estimated glomerular filtration rate (eGFR) < 60 mL/min/1.73 m^2^ for more than 3 months; (3) renal biopsy consistent with DKD pathological changes. The study was approved by the ethics committee of the Affiliated Hospital of Medical School, Ningbo University (KY20171112), and written informed consent was obtained from all participants.

Inclusion criteria: T2DM; age ≥18 years; clearly diagnosed DKD. exclusion criteria: other renal diseases; severe life-threatening organ dysfunction of the heart, lungs, kidney and liver; tumors; hormone use within the past 6 months.

### Procedure

The demographic and clinical data for this study were primarily information that was readily available, relatively complete, and comparable in clinical practice, which was collected through a questionnaire. All staffs involved in the questionnaire received standardized training. The questionnaire survey collected participants' general characteristics [gender, age, family history of DM, duration of T2DM, body mass index (BMI), systolic blood pressure (SBP), diastolic blood pressure (DBP)], biochemical indicators in the last 3 months [glycated hemoglobin A1c (HbA1c), fasting blood glucose (FBG), postprandial (2 h) blood glucose (PBG), triglycerides (TG), total cholesterol (TC), high-density lipoprotein (HDL), low-density lipoprotein (LDL), and UA], and chronic complications. Biochemical indicators were obtained from each hospital's electronic laboratory record system. Confirmation of chronic complications required a review of medical history and inquiry and was recorded only if there was a clear previous diagnosis.

### Statistical analysis

Six-hundred ten patients with DKD were enrolled in this research, and data information for all variables was expressed as counts (%). Statistical analysis was performed with R software (version 4.1.2; https://www.R-project.org). Comparison of the count data between the two groups was performed by chi-square test. All tests were two-tailed, and a *P* value of < 0.05 was considered statistically significant.

**Figure 1:**
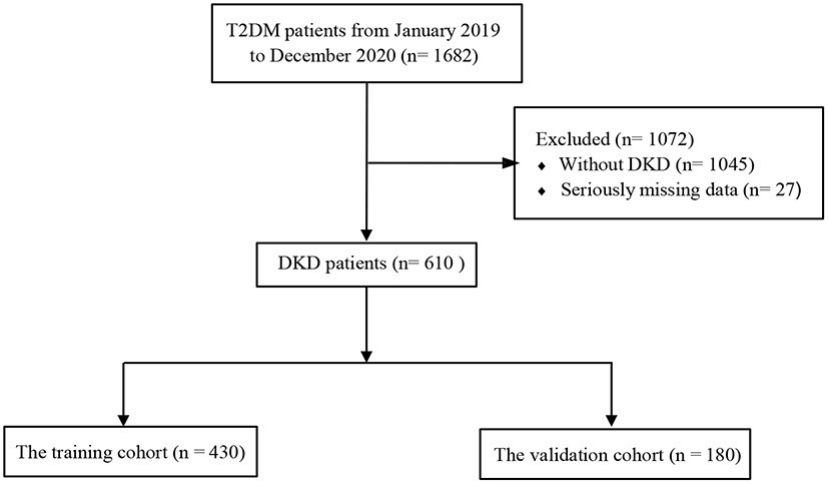
Flowchart of study participants. T2DM, type 2 diabetes mellitus; DKD, diabetic kidney disease.

Participants were randomized to training and validation cohorts according to a certain ratio (7:3) (17, 18), while the random sampling process used the createDataPartition function in the caret package. In addition, we knew from the calculation that the sample size was sufficient for the subsequent statistical analysis, which complied with the rule of 10 events per variable (19, 20). Independent risk factors were identified by multivariable logistic regression analysis. The LASSO is a method applied for data dimensional reduction (21, 22), which could construct a penalty function to obtain a double-standard error. The characteristic variables associated with DKD were screened out by LASSO combined with 10-fold cross-validation. Finally, the HUA risk prediction model is constructed by logistic regression analysis and presented by nomogram (23). The participant screening flow diagram for this study is shown in Figure 1.

The risk predictive models were evaluated in terms of discriminatory ability [C-index and receiver operating characteristic (ROC) curve], calibration ability (Hosmer–Lemeshow test and calibration curve), and clinical applicability [decision curve analysis (DCA)] (17).

## Results

### Characteristics of the research cohort

Six hundred and ten participants were enrolled in this study, including 412 individuals with DKD without HUA and 198 individuals with DKD with HUA. The percentage of HUA in the DKD population was found to be as high as 32.4% in the study. We observed a similar proportion of males in both groups (52.4 vs. 53.5%), an overwhelming majority of age >60 years (73.5 vs. 75.8%), and a predominance of T2DM duration of 15–20 years (34.7 vs. 26.3%). In the DKD population, the HUA group had a higher proportion of smoking history, drinking history, obese patients, FBG >7 mmol/L, HbA1c >8%, hypertension and eGFR ≤ 120 mL/min/1.73m^2^, and hyperlipidemia compared with the control group. BMI (*P* = 0.025), SBP (*P* = 0.004), PBG (*P* = 0.017), HbA1c (*P* < 0.001), UA (*P* < 0.001), eGFR (*P* < 0.001) and hypertension (*P* < 0.001) were found to be significantly different between the two groups by univariate analysis (Table 1). A total of 430 (135 with HUA) and 180 (63 with HUA) participants were assigned to the training and validation cohorts, respectively, by randomization sampling, while it could be seen that the variables did not differ in the training and validation cohorts (Table 1).

**Table 1:** Characteristics of participants in different cohorts.

	**DKD without HUA**	**DKD with HUA**	***P*-Value**	**Training cohort (135 with HUA)**	**Validation cohort (63 with HUA)**	***P*-Value**
N	412	198		430	180	
gender (male), %	216 (52.4)	106 (53.5)	0.863	234 (54.4)	88 (48.9)	0.215
Age, %			0.595			0.859
≤ 50 years old	27 (6.6)	15 (7.6)		29 (6.7)	13 (7.2)	
50–60 years old	82 (19.9)	33 (16.7)		79 (18.4)	36 (20.0)	
> 60 years old	303 (73.5)	150 (75.8)		322 (74.9)	131 (72.8)	
Education level, %			0.712			0.146
Primary/illiterate	283 (68.7)	135 (68.2)		286 (66.5)	132 (73.3)	
Middle/high school	121 (29.4)	61 (30.8)		138 (32.1)	44 (24.4)	
University and above	8 (1.9)	2 (1.0)		6 (1.4)	4 (2.2)	
Family history of T2DM, %	86 (20.9)	41 (20.7)	0.999	89 (20.7)	38 (21.1)	0.913
Smoking history, %	92 (22.3)	52 (26.3)	0.309	106 (24.7)	38 (21.1)	0.403
Drinking history, %	67 (16.3)	38 (19.2)	0.363	76 (17.7)	29 (16.1)	0.724
Duration of T2DM, %			0.070			0.121
≤ 5 years	8 (1.9)	5 (2.5)		9 (2.1)	4 (2.2)	
5–10 years	138 (33.5)	60 (30.3)		131 (30.5)	67 (37.2)	
10–15 years	73 (17.7)	48 (24.2)		88 (20.5)	33 (18.3)	
15–20 years	143 (34.7)	52 (26.3)		149 (34.7)	46 (25.6)	
> 20 years	50 (12.1)	33 (16.7)		53 (12.3)	30 (16.7)	
BMI, %			0.025			0.353
≤ 24 kg/m^2^	184 (44.7)	69 (34.8)		175 (40.7)	78 (43.3)	
24–28 kg/m^2^	171 (41.5)	88 (44.4)		190 (44.2)	69 (38.3)	
> 28 kg/m^2^	57 (13.8)	41 (20.7)		65 (15.1)	33 (18.3)	
SBP, %			0.004			0.677
≤ 140 mmHg	269 (65.3)	101 (51.0)		256 (59.5)	114 (63.3)	
140–160 mmHg	98 (23.8)	73 (36.9)		126 (29.3)	45 (25.0)	
160–180 mmHg	40 (9.7)	21 (10.6)		43 (10.0)	18 (10.0)	
> 180 mmHg	5 (1.2)	3 (1.5)		5 (1.2)	3 (1.7)	
DBP, %			0.562			0.428
≤ 90 mmHg	388 (94.2)	183 (92.4)		398 (92.6)	173 (96.1)	
90–100 mmHg	16 (3.9)	12 (6.1)		23 (5.3)	5 (2.8)	
100–110 mmHg	6 (1.5)	3 (1.5)		7 (1.6)	2 (1.1)	
>110 mmHg	2 (0.5)	0 (0.0)		2 (0.5)	0 (0.0)	
FBG, %			0.633			0.754
≤ 7 mmol/L	174 (42.2)	91 (46.0)		191 (44.4)	74 (41.1)	
7–11 mmol/L	209 (50.7)	96 (48.5)		211 (49.1)	94 (52.2)	
>11 mmol/L	29 (7.0)	11 (5.6)		28 (6.5)	12 (6.7)	
PBG, %			0.017			0.628
≤ 11 mmol/L	128 (31.1)	85 (42.9)		147 (34.2)	66 (36.7)	
11–15 mmol/L	185 (44.9)	75 (37.9)		182 (42.3)	78 (43.3)	
>15 mmol/L	99 (24.0)	38 (19.2)		101 (23.5)	36 (20.0)	
HbA1c, %			< 0.001			0.372
≤ 8%	152 (36.9)	110 (55.6)		179 (41.6)	83 (46.1)	
8–10%	143 (34.7)	58 (29.3)		149 (34.7)	52 (28.9)	
>10%	117 (28.4)	30 (15.2)		102 (23.7)	45 (25.0)	
TC (>6.2 mmol/L), %	37 (9.0)	16 (8.1)	0.761	35 (8.1)	18 (10.0)	0.528
TG (>4.1 mmol/L), %	71 (17.2)	43 (21.7)	0.185	81 (18.8)	33 (18.3)	0.910
LDL (>3.4 mmol/L), %	68 (16.5)	22 (11.1)	0.088	66 (15.3)	24 (13.3)	0.617
HDL (>1 mmol/L), %	265 (64.3)	114 (57.6)	0.110	271 (63.0)	108 (60.0)	0.522
UA, %			< 0.001			0.890
≤ 360 μmol/L	325 (78.9)	17 (8.6)		245 (57.0)	97 (53.9)	
360–420 μmol/L	73 (17.7)	37 (18.7)		76 (17.7)	34 (18.9)	
420–480 μmol/L	10 (2.4)	49 (24.7)		40 (9.3)	19 (10.6)	
>480 μmol/L	4 (1.0)	95 (48.0)		69 (16.0)	30 (16.7)	
eGFR, %			< 0.001			0.164
>120, mL/min/1.73 m^2^	320 (77.7)	70 (35.4)		269 (62.6)	121 (67.2)	
90–120, mL/min/1.73 m^2^	55 (13.3)	39 (19.7)		76 (17.7)	18 (10.0)	
60–90, mL/min/1.73 m2	25 (6.1)	46 (23.2)		49 (11.4)	22 (12.2)	
30–60, mL/min/1.73 m^2^	7 (1.7)	34 (17.2)		27 (6.3)	14 (7.8)	
≤ 30, mL/min/1.73 m^2^	5 (1.2)	9 (4.5)		9 (2.1)	5 (2.8)	
Hypertensive, %	310 (75.2)	179 (90.4)	< 0.001	356 (82.8)	133 (73.9)	0.014
Hyperlipidemia, %	204 (49.5)	115 (58.1)	0.057	227 (52.8)	92 (51.1)	0.723
Atherosclerosis, %	394 (95.6)	187 (94.4)	0.545	405 (94.2)	176 (97.8)	0.062
CVD, %	103 (25.0)	54 (27.3)	0.554	109 (25.3)	48 (26.7)	0.761

DKD, diabetic kidney disease; HUA, hyperuricemia; BMI, body mass index; SBP, systolic blood pressure; DBP, diastolic blood pressure; FBG, fasting blood glucose; PBG, postprandial (2 h) blood glucose; TC, total cholesterol; TG, triglycerides; LDL, low–density lipoprotein; HDL, high–density lipoprotein; UA, uric acid; eGFR, estimated glomerular filtration rate; CVD, cardiovascular disease.

### Independent risk factors

These variables were incorporated into multivariate logistic regression analyses according to the results of the univariate analysis in Table 1 (with a screening criterion of *P* < 0.1). BMI, HbA1c, eGFR, and hyperlipidemia were identified as independent risk factors for HUA in the DKD population (Table 2).

**Table 2:** Multivariate logistic regression analysis.

**Variable**	**Coefficients**	**Odds Ratio (95% CI)**	***P-*value**
BMI			
≤ 24 kg/m^2^		1	1
24–28 kg/m^2^	0.441	1.554 (1.002–2.426)	< 0.05
>28 kg/m^2^	0.83	2.294 (1.310–4.021)	< 0.01
HbA1c			
≤ 8%		1	1
8–10%	−0.406	0.666 (0.424–1.041)	>0.05
>10%	−0.707	0.493 (0.287–0.834)	< 0.01
eGFR			
>120 mL/min/1.73 m^2^		1	1
90–120 mL/min/1.73 m^2^	1.200	3.322 (1.996–5.528)	< 0.01
60–90 mL/min/1.73 m^2^	2.144	8.541 (4.859–15.367)	< 0.01
30–60 mL/min/1.73 m^2^	3.211	24.814 (10.849–64.722)	< 0.01
≤ 30 mL/min/1.73 m^2^	2.221	9.214 (2.892–32.562)	< 0.01
Hyperlipidemia	0.600	1.822 (1.216–2.758)	< 0.01

BMI, body mass index; eGFR, estimated glomerular filtration rate.

### Construction of predictive models

**Figure 2:**
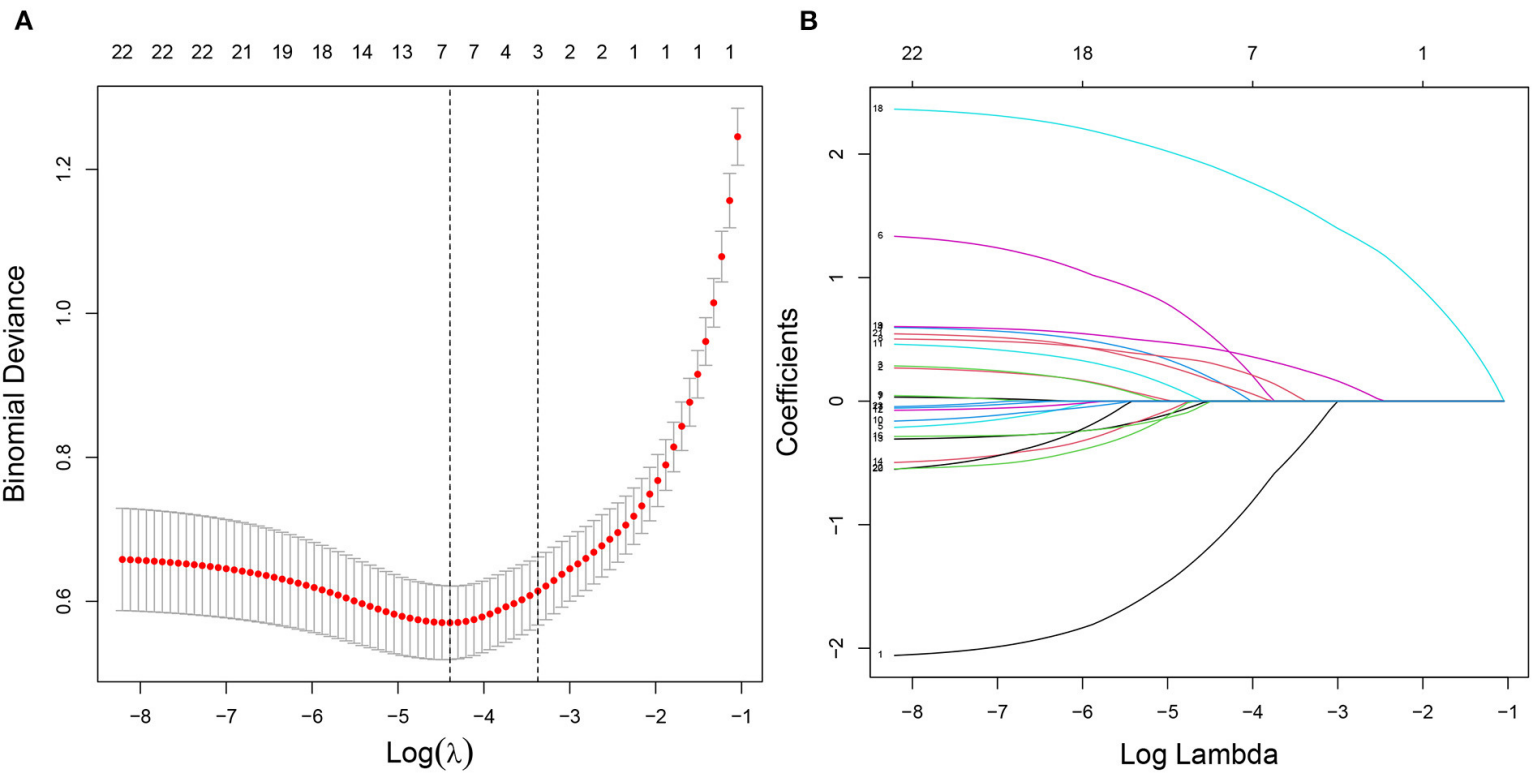
Characteristic variables were screened using LASSO regression analysis. **(A)** The selection of the best parameter (lambda) in the LASSO model uses 10-fold cross-validation with the lowest standard. The relationship curve between partial likelihood deviation (binomial deviation) and log (lambda) was plotted. Dotted vertical lines were drawn at the optimal values by using the minimum criteria and the one SE of the minimum criteria (the one SE criteria). **(B)** LASSO coefficient profiles of the seven characteristic variables. A coefficient profile plot was produced against the log (lambda) sequence. LASSO, least absolute shrinkage and selection operator; SE, standard error.

**Figure 3:**
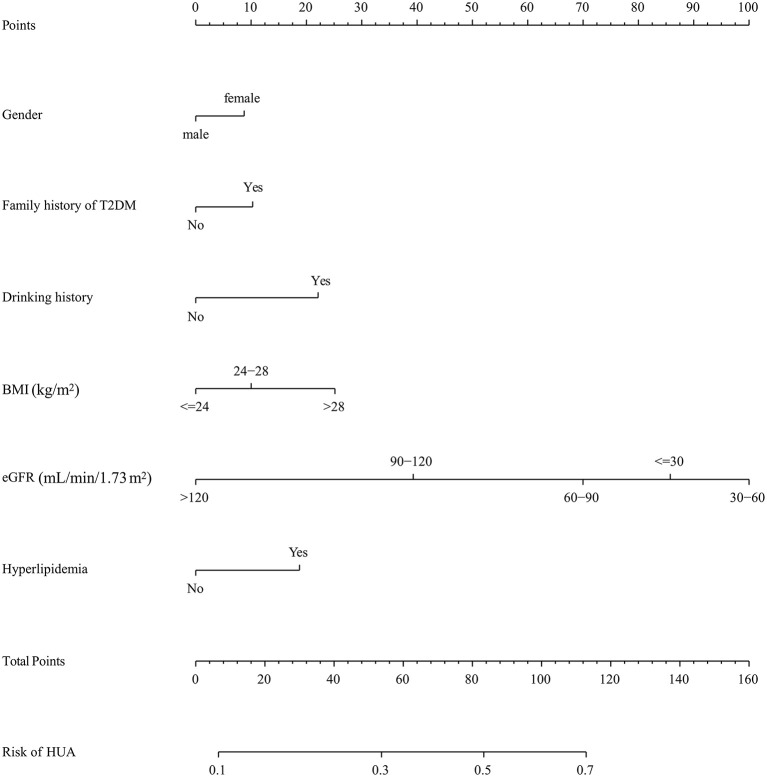
A nomogram for predicting the probability of developing HUA in DKD population. The nomogram is used by scoring each variable on its corresponding score scale. The scores for all variables are then summed to obtain the total score, and a vertical line is drawn from the total point row to indicate the estimated probability of the development of HUA in DKD population. DKD, diabetic kidney disease; BMI, body mass index; eGFR, estimated glomerular filtration rate.

In the training cohort, seven nonzero characteristic variables, such as gender, family history of T2DM, drinking history, BMI, UA, eGFR, and hyperlipidemia, were screened out by LASSO combined with 10-fold cross-validation (Figure 2; Table 3). Since UA is one of the diagnostic criteria for HUA, we selected gender, family history of T2DM, drinking history, BMI, eGFR, and hyperlipidemia as predictors to construct the HUA risk model by logistic regression analysis, which was visualized by nomogram (Figure 3).

**Table 3:** Coefficients and lambda.min value of the LASSO regression.

**Variables**	**Coefficients**	**Lambda.min**
Gender	−1.106	0.012
Family history of T2DM	0.154	
Drinking history	0.475	
BMI	0.291	
UA	1.879	
eGFR	0.416	
Hyperlipidemia	0.147	

BMI, body mass index; UA, uric acid; eGFR, estimated glomerular filtration rate.

### Validation of predictive models

**Figure 4:**
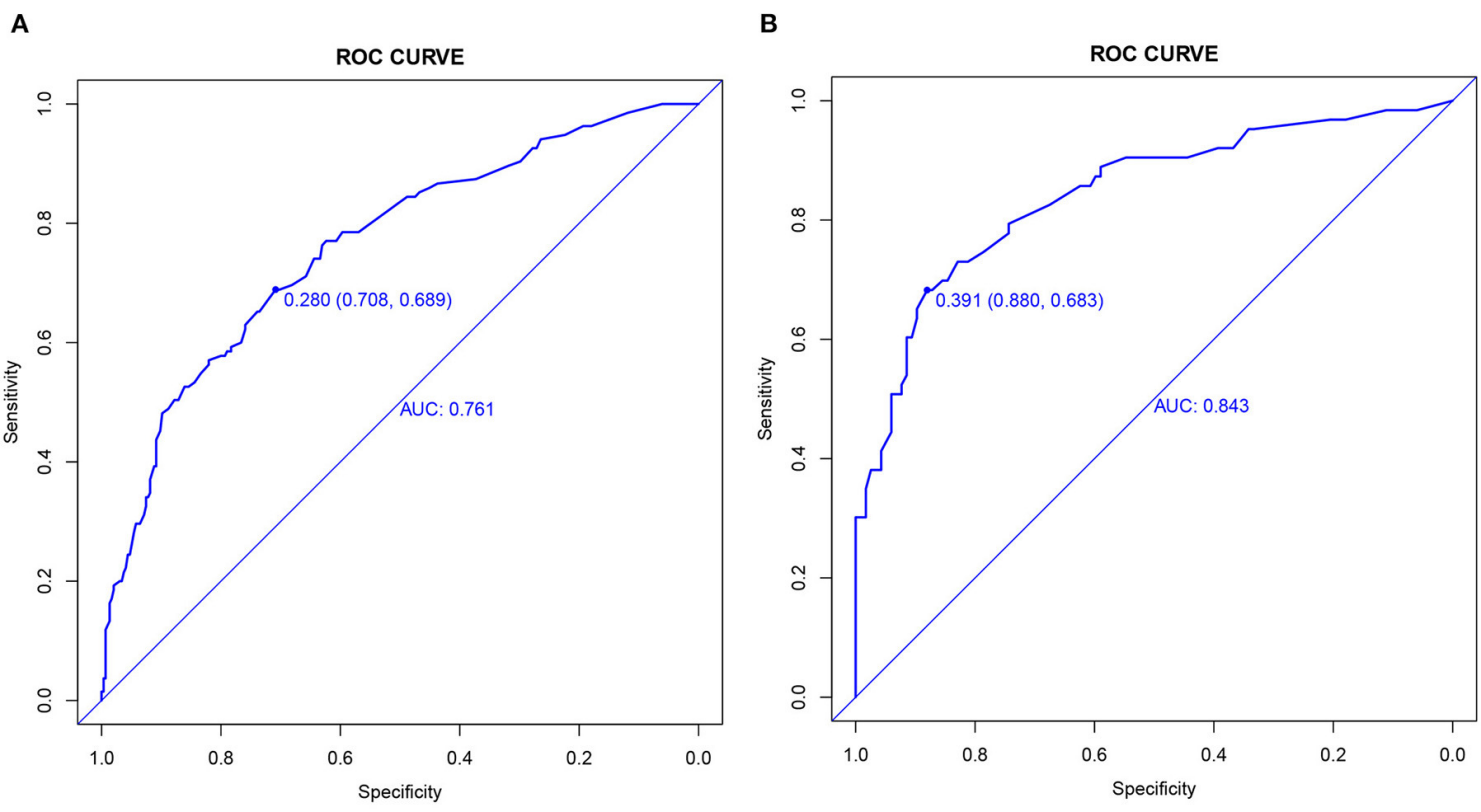
ROC curves**. (A)** Training cohort and **(B)** validation cohort.

The C-index and the area under the ROC curve (AUC) were used to assess the discriminatory ability of the risk model. In the training cohort, the C-index was 0.761 (95% CI: 0.712–0.810), and the AUC was 0.761, while in the validation cohort, the values were 0.843 (95% CI: 0.780–0.906) and 0.843 (Figure 4).

**Figure 5:**
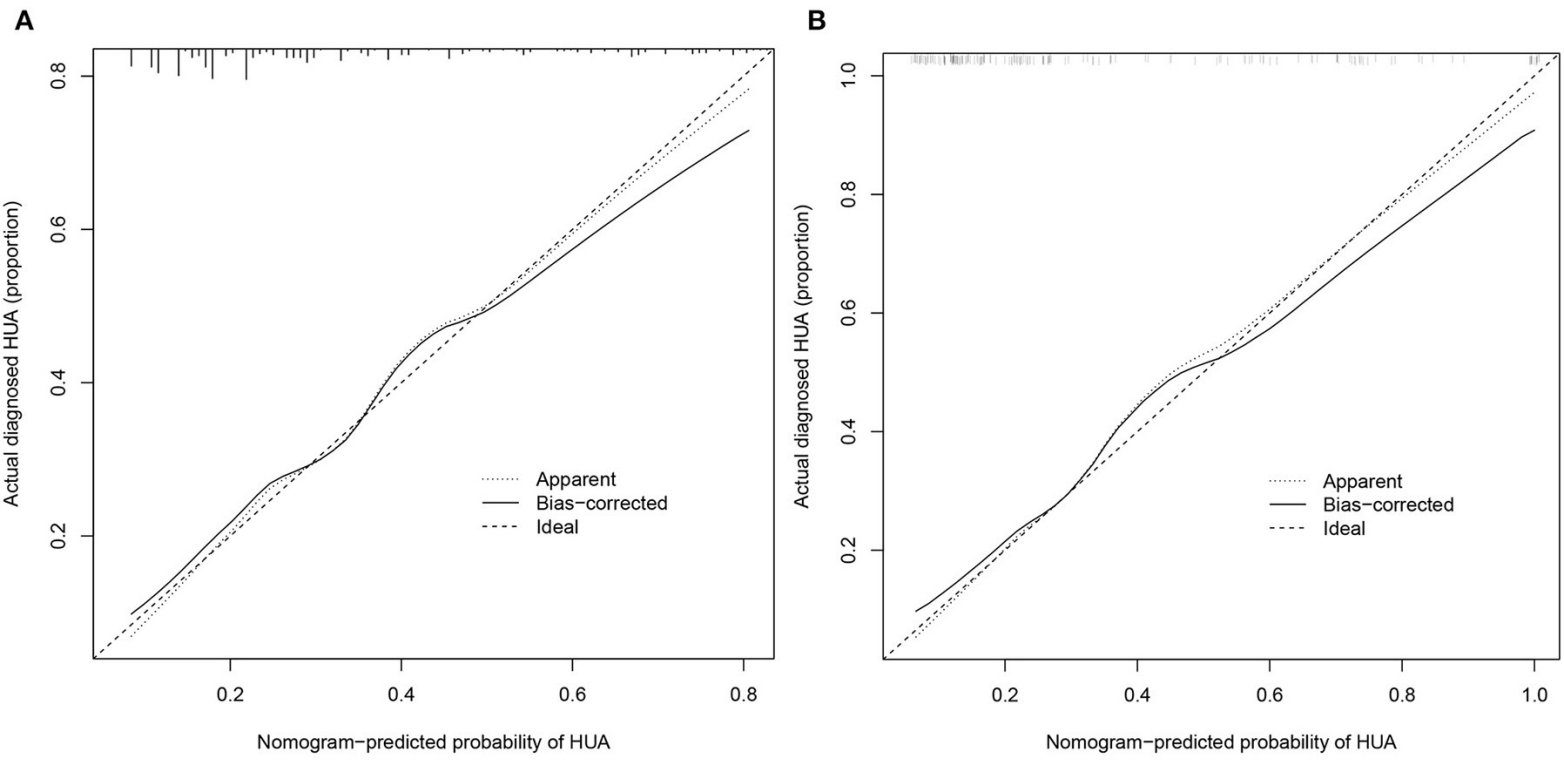
Calibration curves**. (A)** Training cohort and **(B)** validation cohort. The x-axis represents the predicted HUA risk. The y-axis represents the actual diagnosed HUA. The diagonal dotted line represents a perfect prediction by an ideal model. The solid line represents the performance of the nomogram, of which a closer fit to the diagonal dotted line represents a better prediction.

From the calibration curve, the predicted values were very close to the theoretical values in the training and validation cohorts, showing an excellent degree of fit (Figure 5), which was further confirmed by the Hosmer–Lemeshow test (*P* > 0.05) (Table 4).

**Table 4:** Hosmer–Lemeshow test.

	**χ^2^**	***P*-Value**
Training cohort	3.696	0.930
Validation cohort	7.456	0.590

**Figure 6:**
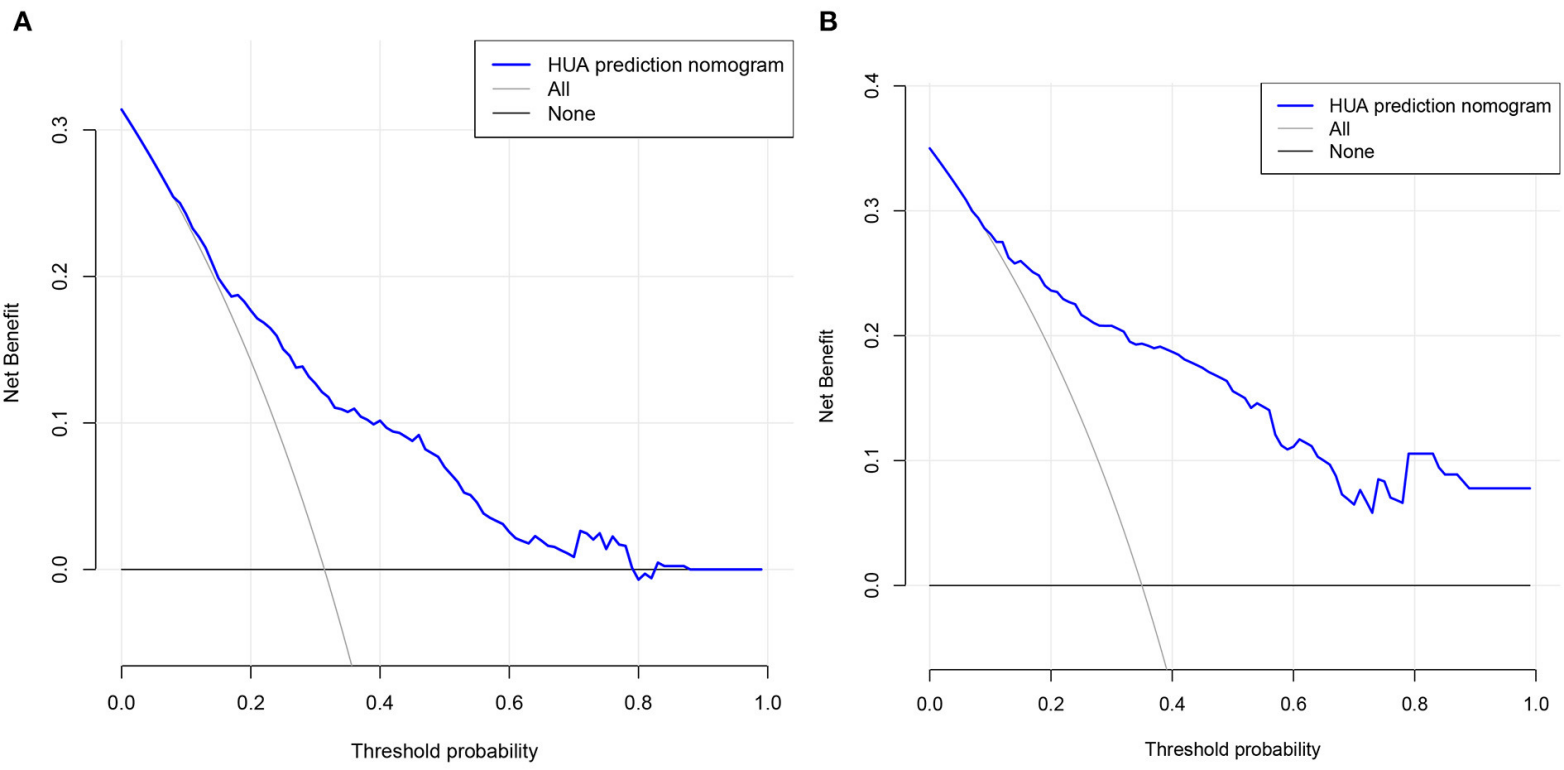
Decision curve analysis. **(A)** Training cohort and **(B)** validation cohort. The black line represents the net benefit when none of the participants are considered to develop HUA, while the light gray line represents the net benefit when all participants are considered to develop HUA. The area between the blue line and light gray line in the model curve indicates the clinical utility of the model.

DCA is a method that has been used to evaluate the clinical applicability of risk models. Figure 6 shows that the risk threshold probabilities for the training and validation cohorts were 9–79% and 7–100%, respectively, which suggested that the risk prediction model could benefit patients within this threshold probability range.

## Discussion

DKD seriously affects the quality of life of T2DM patients and threatens their lives, while an increasing number of scholars have started to pay attention to and study the relationship between UA and DKD (24–26). Through a retrospective investigation in Ningbo, China, 610 DKD patients were enrolled, including 198 HUA patients. The multivariate logistic regression analysis identified BMI, HbA1c, eGFR, and hyperlipidemia as independent risk factors for HUA in the DKD population. The characteristic variables, such as gender, family history of T2DM, drinking history, BMI, eGFR, and hyperlipidemia, were screened as predictors for the HUA risk model by LASSO combined with 10-fold cross-validation. We then validated the risk prediction model in terms of discrimination, fitting degree, and clinical applicability. In the training and validation cohorts, the C-index was 0.761 (95% CI: 0.712–0.810) and 0.843 (95% CI: 0.780–0.906), respectively; the DCA showed that the participants could benefit when the risk probability thresholds were 9–79% and 7–100%; meanwhile, the risk model passed the Hosmer–Lemeshow test with a high goodness of fit.

The proportion of HUA among DKD patients was found to be 32.4% in the study, higher than the 13% in Zhengzhou, China (27), which might be related to the region as well as the inclusion of the study population. Current research in this area is still limited and more studies are necessary in the future. The relationship between DKD and HUA is complex, causally indistinguishable, and mutually reinforcing (28), and the prevailing view is that UA is a modifiable and independent risk factor for chronic kidney disease (29). In contrast, we identified independent risk factors associated with HUA based on the DKD population. Obesity as a risk factor for HUA has been proven in several studies (30–32). The accumulation of visceral fat in obese people affects the metabolic capacity of the kidneys, thus inhibiting the excretion of UA (33, 34). Our research showed that hyperlipidemia is a risk factor for HUA, which was supported by a previous study (35). Although the cause of the increased prevalence of HUA due to lipid metabolism is unknown, potential mechanisms may be related to the metabolic pathways of free fatty acids (36). A chronic hyperglycemic state stimulates the pancreas to produce insulin overload, and elevated insulin promotes UA reabsorption by the proximal renal tubules (37). Therefore, a higher HbA1c often means a higher incidence of HUA (38). In addition, DKD patients already have impaired renal excretion performance, which, together with the above risk factors, would further increase the elevation of UA.

The construction of predictive models is important for the early diagnosis and prevention of diseases. Various HUA risk prediction models have been established in recent years based on normal populations in different regions (11–13, 39), all showing good clinical differentiation. However, the available HUA risk models still have some limitations. Although Cox regression models, artificial neural networks, and random forest models demonstrate good clinical predictive value, the clinical applicability is limited due to their low visualization. Nomograms are often used to visualize risk prediction models due to their simplicity, visualization, and operability. It mainly assigns a value to each predictor based on the regression coefficient and uses the corresponding algorithm to derive a predictive value for the corresponding individual outcome event (40). In addition, previous studies have revealed that the nomogram model outperforms other machine learning models (artificial neural networks and classification tree models) in accuracy and clinical utility (41, 42). In this study, LASSO combined with 10-fold cross-validation screened for characteristic variables associated with HUA, such as gender, family history of T2DM, drinking history, BMI, eGFR, and hyperlipidemia, which are the most readily available variables in clinical practice. The establishment of visual predictive models can better contribute to the early diagnosis and prevention of HUA in the DKD population, which is of great significance for countries or regions with relatively scarce medical resources.

Compared to previous studies, we have the following advantages. First, we identified risk factors associated with HUA based on the DKD population, which has rarely been reported. HUA is well known as a risk factor for DKD, while the identification of HUA risk factors could help delay the progression of DKD and reduce the occurrence of adverse prognostic events. Second, we may be the first to develop an HUA risk prediction model based on a DKD population, which has important implications for the early diagnosis and prevention of the disease. Certainly, there are some limitations in our study. First, the diagnosis of DKD is predominantly clinical, so the presence of nondiabetic kidney disease cannot be completely ruled out. Second, as a cross-sectional study, there is no escape from the fact that our sample size was limited. Third, the HUA risk prediction model is only validated by internal datasets, while the validation of external datasets is necessary. Furthermore, we will expand the sample size to improve the stability of the model; meanwhile, we will cooperate with multiple centers to obtain external datasets to validate the model.

## Conclusions

Briefly, based on a multicenter study in Ningbo, China, we identified independent risk factors (BMI, SBP, eGFR, and hyperlipidemia) associated with HUA and constructed an HUA risk prediction model in the DKD population. The establishment of risk prediction helps us to identify individuals at high risk of HUA early in the DKD population, which is important for the prevention and reduction of adverse prognostic events in DKD.

## Data availability statement

The original contributions presented in this study are included in the article, further inquiries can be directed to the corresponding author/s.

## Ethics statement

The study was reviewed and approved by the Ethics Committee of the Affiliated Hospital of Medical School, Ningbo University, Ningbo, China. The patients/participants provided their written informed consent to participate in this study.

## Author contributions

GH and ML conceived and designed the research, drafted the manuscript, and took part in the discussion. GH performed the statistical analysis. YM and YL revised the manuscript. All authors contributed to the article and approved the submitted version.

## Funding

This research was supported by grants from Natural Science Fund of Ningbo, China (No. 2018A610248), NINGBO Medical & Health Leading Academic Discipline Project (No. 2022-F24), Zhejiang Medicine and Health Technology Project, China (No. 2018ZH029 and 2020KY871), the Major Project for Science and Technology Innovation 2025 in Ningbo, China (No. 2019B10035), Ningbo Social Development, China (No. 2019C50080), and Ningbo Social Welfare Research (No. 2022S047).

## Conflict of interest

The authors declare that the research was conducted in the absence of any commercial or financial relationships that could be construed as a potential conflict of interest.

## Publisher's note

All claims expressed in this article are solely those of the authors and do not necessarily represent those of their affiliated organizations, or those of the publisher, the editors and the reviewers. Any product that may be evaluated in this article, or claim that may be made by its manufacturer, is not guaranteed or endorsed by the publisher.
